# Supplementary material for: Importance of localized dilatation and distensibility in identifying determinants of thoracic aortic aneurysm with neural operators
Source: PLoS Comput Biol. 2025 Oct 9;21(10):e1013550. doi: 10.1371/journal.pcbi.1013550 (PMC12520381; doi:10.1371/journal.pcbi.1013550)
Supplement: S1 — Supplemental Tables A & B and Figs A–G. (PDF) [file pcbi.1013550.s001.pdf]

Importance of localized dilatation and distensibility in identifying determinants of thoracic aortic aneurysm with neural operators

Supporting Information

David S. Li[a], Somdatta Goswami[b], Qianying Cao[c], Vivek Oommen[d], Roland Assi[e], Jay D. Humphrey[a,f], George E. Karniadakis[c]

[a]*Department of Biomedical Engineering, Yale University*
[b]*Department of Civil and Systems Engineering, Johns Hopkins University*
[c]*Division of Applied Mathematics, Brown University*
[d]*School of Engineering, Brown University*
[e]*Department of Surgery, Yale School of Medicine*
[f]*Vascular Biology and Therapeutics Program, Yale School of Medicine*

Constitutive relations

We employed a well-established framework for aortic growth and remodeling (G&R) based on the notion of mechanobiological homeostasis in a healthy aorta in maturity [1], wherein aortic cells modulate the production and removal of extracellular matrix components in response to perturbations in order to maintain intramural stress near homeostatic "set-points" σ_o [2] The aortic wall was modeled as a constrained mixture of constituents α defining its material properties, namely elastin-dominated matrix ($\alpha = e$), smooth muscle cells ($\alpha = m$), and collagen-dominated matrix ($\alpha = c$). Importantly, the latter two constituents are capable of turnover via growth (changes in mass) and remodeling (changes in structure) in response to deviations in stress from normal $\Delta\sigma = ((1-\delta)\sigma-\sigma_o)/\sigma_o$, where δ captures mechanosensing capability and $\sigma = \operatorname{tr} \boldsymbol{\sigma}/3$ is a scalar representation of the current intramural (Cauchy) stress defined as

$$\boldsymbol{\sigma} = 2\mathbf{F}(\partial W/\partial \mathbf{C})\mathbf{F}^{\mathrm{T}}/\det \mathbf{F}, \tag{1}$$

with $\mathbf{F}$ the deformation gradient tensor, $\mathbf{C}$ the right Cauchy-Green tensor, and W the constitutive relation for total stored energy. W may be defined conceptually as a sum of constituent-specific stored energies ($W = \sum_{\alpha=e,c,m} \phi^\alpha \hat{W}^\alpha$), which we defined separately for elastin and smooth muscle cells and collagen as

$$\hat{W}^e = c^e(I_1^e - 3) \quad \hat{W}^{m,c} = \frac{c_1^{m,c}}{4c_2^{m,c}}\Big(\exp\big(c_2^{m,c}(I_4^{m,c}-1)^2\big) - 1\Big) \tag{2}$$

with associated mass fractions ϕ^α.

In this study, we assumed that the stimulus functions were not responsive to changes in wall shear stress owing to both endothelial dysfunction and smooth muscle phenotypic modulation associated with thoracic aortic aneurysm (TAA). Geometric, material, and turnover parameters used in this study are summarized in Table A.

Table A: Baseline material and G&R parameters. Superscripts e, m, c denote elastin-dominated matrix, smooth muscle, and collagen-dominated matrix, respectively; super/-subscripts r, θ, z, d denote radial, circumferential, axial, and symmetric diagonal directions, respectively. Data are based on studies of mouse models of thoracic aortopathy [3, 4].

	Parameter	Value
Inner radius, thickness, length	r_o, h_o, l_o	0.808 mm, 0.041 mm, 10 mm
Elastin, smooth muscle, collagen mass fractions	$\phi_o^e, \phi_o^m, \phi_o^c$	0.354, 0.298, 0.348
Collagen orientation fractions	$\beta^\theta, \beta^z, \beta^d$	0.077, 0.035, 0.888
Diagonal collagen orientation	α_{0o}	50.3°
Elastin material parameters	c^e	92.6 kPa
Smooth muscle material parameters	c_1^m, c_2^m	0.32 kPa, 31.1
Collagen material parameters	c_1^c, c_2^c	4446.5 kPa, 2.45
Elastin deposition stretches	G_r^e, G_θ^e, G_z^e	$1/(G_\theta^e G_z^e)$, 2.11, 2.03
Smooth muscle and collagen deposition stretches	G^m, G^c	1.20, 1.06
Mechanosensing	δ	0
Smooth muscle-to-collagen turnover ratio	η	1.0
Shear-to-intramural gain ratio	K_{τ_w}/K_σ	0.2

Insult profiles generated via Gaussian random fields

As described previously [5], we used Gaussian random fields (GRFs) to define randomly distributed insult profiles throughout the aortic (z–θ) domain. Latent profiles ϑ^* were sampled according to

$$\vartheta^*(z_o, \theta_o) \sim \mathcal{G}\big(\mu(z_o, \theta_o), \kappa(z_o, \theta_o, z_o', \theta_o')\big), \tag{3}$$

where $\mu(z_o, \theta_o)$ and $\kappa(z_o, \theta_o, z_o', \theta_o')$ are the mean and covariance between points (z_o, θ_o) and (z_o', θ_o'), with o denoting the reference configuration. The mean and covariance can be controlled by the user to specify the overall approximate insult surface fraction φ, boundary softness of the insult region ϵ, and length scale along the circumferential and axial directions, L_θ and L_z, respectively. The mean and variance of the GRF were given by

$$\begin{aligned} \mu &= \frac{1}{2} - \frac{1}{\epsilon\sqrt{\pi}} \operatorname{erf}^{-1}(1-2\varphi) \exp\left(-\left[\operatorname{erf}^{-1}(1-2\varphi)\right]^2\right) \\ \text{and} \quad \varsigma^2 &= \frac{1}{2\pi\epsilon^2} \exp\left(-2\left[\operatorname{erf}^{-1}(1-2\varphi)\right]^2\right), \end{aligned} \tag{4}$$

where $\operatorname{erf}^{-1}(\cdot)$ is the inverse of the error function. μ is assumed to be constant with respect to z_o and θ_o. The insult propensity φ corresponds to the fraction of ϑ^* values greater than 0.5, and ϵ corresponds to the slope of the cumulative distribution function of ϑ^* when $\vartheta^* = 0.5$. The covariance function, enforced to be periodic in θ, was defined as

$$
\begin{aligned}
&\kappa(z_o,\theta_o,z_o',\theta_o') = \varsigma^2 \exp\left(-\frac{1}{2}\left[\left(\frac{D_\theta\left(\theta_o,\theta_o'\right)}{L_\theta}\right)^2 + \left(\frac{D_z\left(z_o,z_o'\right)}{L_z}\right)^2\right]\right)\\
&\text{with}\quad D_\theta\left(\theta_o,\theta_o'\right) = 2r_o \sin\left(\frac{1}{2}\left|\theta_o-\theta_o'\right|\right) \quad\text{and}\quad D_z\left(z_o,z_o'\right) = \left|z_o-z_o'\right|.
\end{aligned}
\tag{5}
$$

When mapping ϑ^* to the vessel wall nodes in the finite element simulations, a multivariate Gaussian distribution $\mathcal{N}$ with mean vector $\boldsymbol{\mu} = \mu\mathbf{1}$ and covariance matrix $\boldsymbol{\Sigma}$ was adopted, where $\Sigma_{ij} = \Sigma_{ji} = \kappa(z_{o,i},\theta_{o,i},z_{o,j},\theta_{o,j})$. Partitioning the mesh into the set of interior nodes a and the set of boundary nodes b, we conditioned the distribution of ϑ_a^* on the enforced value of ϑ_b^* using

$$
\begin{aligned}
\boldsymbol{\mu}_a' &= \boldsymbol{\mu}_a + \boldsymbol{\Sigma}_{ab}\boldsymbol{\Sigma}_{bb}^{-1}\left(\vartheta_b^*\mathbf{1} - \boldsymbol{\mu}_b\right) = \mu + \boldsymbol{\Sigma}_{ab}\boldsymbol{\Sigma}_{bb}^{-1}\left(\vartheta_b^* - \mu\right)\mathbf{1}\\
\boldsymbol{\Sigma}_{aa}' &= \boldsymbol{\Sigma}_{aa} - \boldsymbol{\Sigma}_{ab}\boldsymbol{\Sigma}_{bb}^{-1}\boldsymbol{\Sigma}_{ba}\\
\boldsymbol{\mu}_b' &= \vartheta_b^*\mathbf{1}\\
\boldsymbol{\Sigma}_{ab}' &= \mathbf{0}, \quad \boldsymbol{\Sigma}_{ba}' = \mathbf{0}, \quad \boldsymbol{\Sigma}_{bb}' = \mathbf{0}.
\end{aligned}
\tag{6}
$$

After ϑ_i^* is sampled from $\mathcal{N}\left(\boldsymbol{\mu}',\boldsymbol{\Sigma}'\right)$, a cumulative distribution function (CDF)/inverse-CDF transformation was performed so that the overall distribution of ϑ^* values in each random instance of ϑ_i^* matches the desired $\mathcal{N}\left(\mu,\varsigma^2\right)$. Specifically,

$$
\left(\vartheta_i^*\right)' = \Phi^{-1}\left(F\left(\vartheta_i^*\right);\mu,\varsigma^2\right), \tag{7}
$$

where F is the CDF of the generated random field values and Φ^{-1} is the inverse CDF of the normal distribution with mean μ and variance ς^2. Finally, the insult field values were censored using $\vartheta_i = \min\left(\max\left(\left(\vartheta_i^*\right)',0\right),1\right)$, and the resulting profiles corresponded in turn to patterns of compromised elastic fiber integrity and mechanosensing.

Finite element model

The aortic geometry and material properties were based on biaxial mechanical and histological data from a common mouse model of Marfan syndrome ($Fbn1^{C1041G/+}$), estimated with previously discussed methods [6]. The aorta was meshed with quadratic hexahedral elements with a radial, circumferential, and axial resolution of $1 \times 20 \times 20$, selected after previous mesh sensitivity studies to achieve sufficient accuracy while balancing computational efficiency. Using a custom plugin in the FEBio solver (`FEBio.org`), G&R of the vessel in response to the combined elastic fiber integrity and mechanosensing insults was computed under constant systolic loading conditions of 120 mmHg over a series of gradual pseudo-time increments representing evolution over the course of weeks. After the final (systolic) geometry was achieved, the G&R was arrested while the internal pressure was adjusted to a diastolic condition of 80 mmHg, a time frame corresponding to that of a cardiac cycle. Dilatation and distensibility quantities were evaluated on a nodal basis, that is, a two-dimensional domain of 41×40 nodes axially and circumferentially (noting periodicity in the circumferential direction). We performed circular padding in the circumferential direction (i.e., appended one

additional column by repeating the first column) to produce input data with dimensions of 41×41.

Region-specific error evaluations

In addition to computing prediction errors across the entire aortic domain, we also evaluated errors in the most affected regions of the vessel, namely any location with a normalized insult value of greater than 50%. The overall errors are summarized in Table B.

Table B: Relative $\mathcal{L}_2$ errors for all network-input data combinations predicting combined insult contributors, evaluated over all testing cases. Errors are evaluated over both the total domain as well as only regions with ≥50% normalized insult. The best results across all combinations (lowest $\mathcal{L}_2$ error) are highlighted by boldface in both approaches.

	Overall Relative $\mathcal{L}_2$ Error: Total Domain / ≥50% Insult			
	d Grayscale	d Heat	d & $\mathcal{D}$ Grayscale	d & $\mathcal{D}$ Heat
**Eln Fiber Integrity**				
CNN-DeepONet	0.0614 / 0.0541	0.0534 / 0.0439	0.0244 / 0.0169	0.0237 / 0.0150
FNN-DeepONet	0.0689 / 0.0636	0.1275 / 0.1183	0.0329 / 0.0278	0.0276 / 0.0202
UNet	0.0560 / 0.0528	0.0542 / 0.0515	**0.0176** / 0.0132	0.0182 / **0.0130**
LNO	0.1708 / 0.1710	0.1584 / 0.1582	0.0263 / 0.0236	0.0223 / 0.0192
**Mechanosensing**				
CNN-DeepONet	0.0738 / 0.0655	0.0596 / 0.0481	0.0284 / 0.0213	0.0257 / 0.0168
FNN-DeepONet	0.0856 / 0.0754	0.1564 / 0.1360	0.0444 / 0.0403	0.0377 / 0.0310
UNet	0.0675 / 0.0633	0.0628 / 0.0596	0.0187 / 0.0152	**0.0182** / **0.0147**
LNO	0.1955 / 0.1952	0.1829 / 0.1819	0.0299 / 0.0269	0.0229 / 0.0200

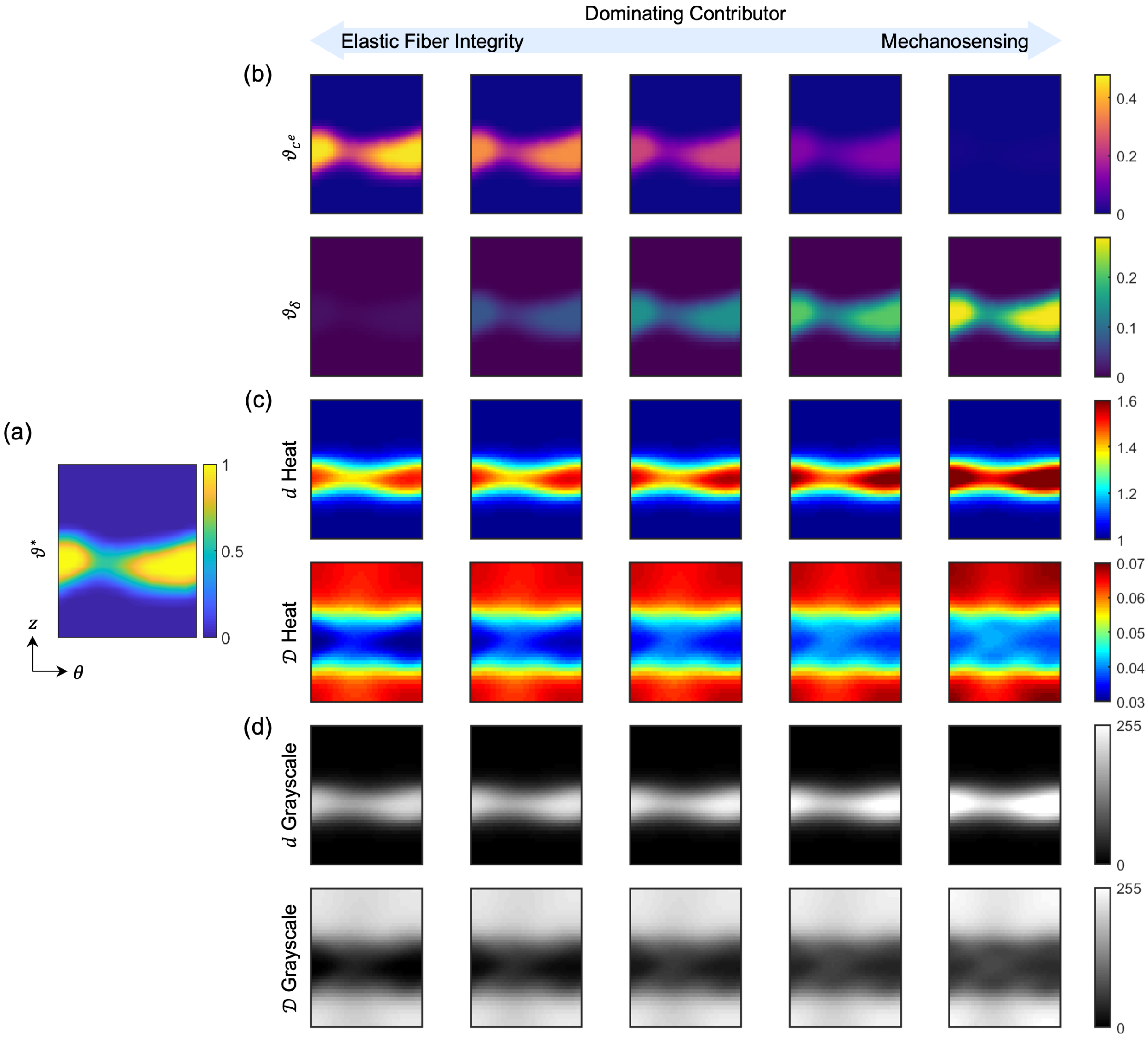

Fig A: Dilatation and distensibility maps. Effects on dilatation d and distensibility $\mathcal{D}$ depending on the dominating contributor (compromised elastic fiber integrity or dysfunctional mechanosensing) in the combined insult. (a) Normalized insult field ϑ^*. (b) Degree of compromised integrity of elastic fibers ($\vartheta_{c^e} \in [0, 0.48]$, decreasing left-to-right) and dysfunctional mechanosensing ($\vartheta_\delta \in [0, 0.28]$, increasing left-to-right) with spatial distributions defined by the normalized insult profile. (c) Heat maps for dilatation and distensibility for each combined insult. (d) Normalized 8-bit grayscale maps.

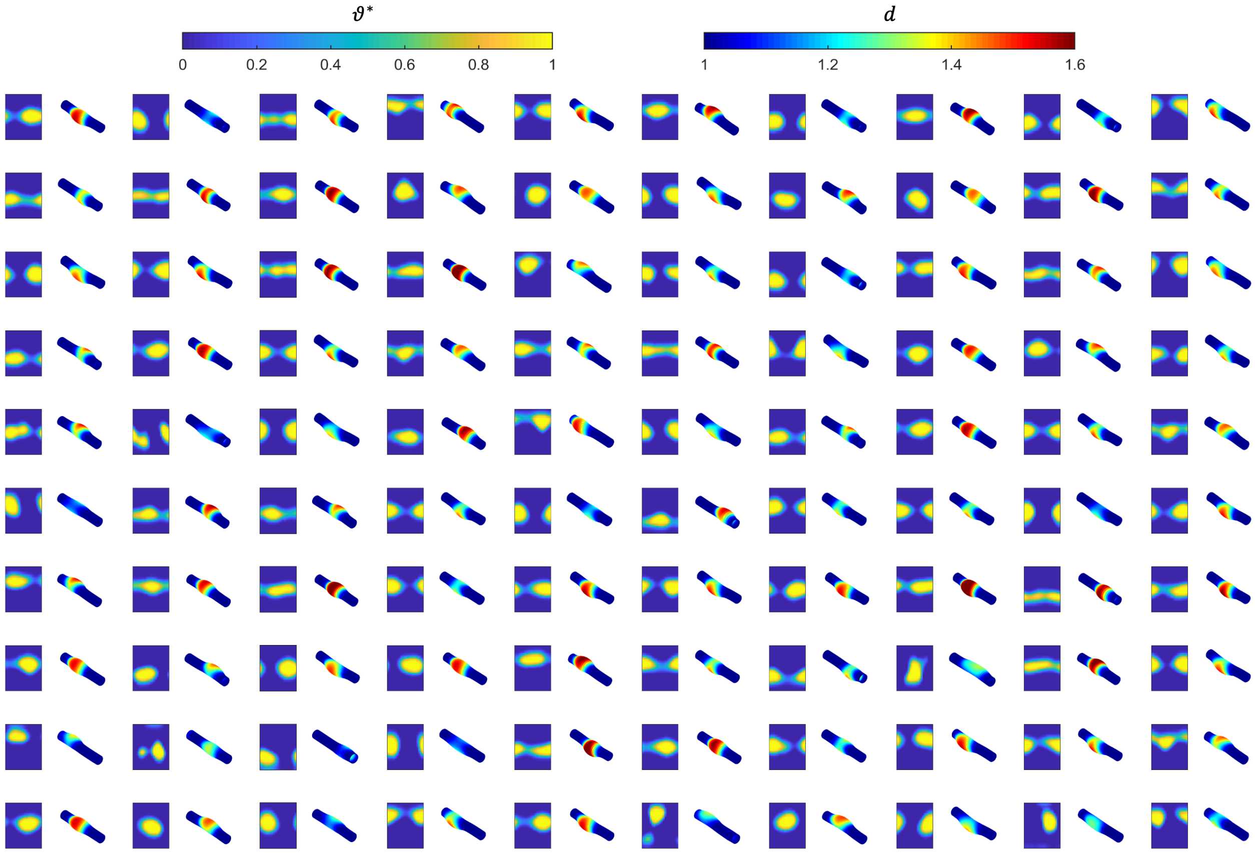

Fig B: Illustrative results. Insult fields (ϑ^*) and resulting TAA geometries colored by dilatation (d). A total of 100 (10×10 panels) unique spatial distributions were generated with Gaussian random fields, and each profile was assigned a random combination of compromised elastic fiber integrity ($\vartheta_{c^e} \in [0, 0.48]$) and dysfunctional mechanosensing ($\vartheta_\delta \in [0, 0.28]$). Shown here is the dilatation corresponding to the most mechanosensing-dominated combination. We set the average circumferential and axial lengths to 4.5 mm and 4.5 mm, respectively, with a boundary softness of 0.2 and an overall insult area of roughly 23% the total vessel area.

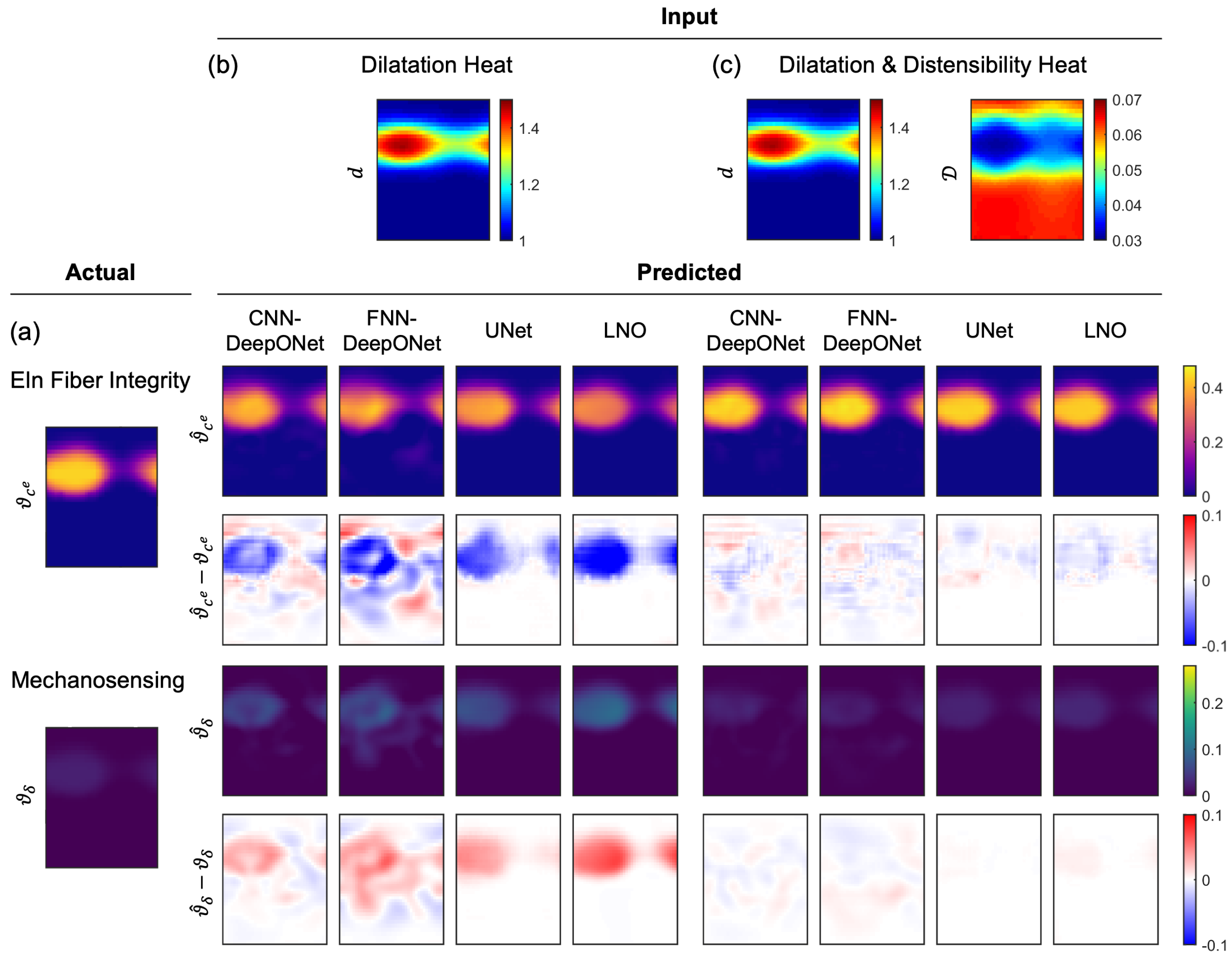

Fig C: Predictions from all four architectures for an elastic fiber integrity-dominated combined insult. (a) The ground truth combined-insult field consisted of contributions of both compromised elastic fiber integrity (ϑ_{c^e}) and dysfunctional mechanosensing (ϑ_δ) in the FE simulation to generate the TAA with dilatation and distensibility profiles shown in (b–c). Predictions ($\hat{\vartheta}_i$) and absolute errors ($\hat{\vartheta}_i - \vartheta_i$ ($i = c^e, \delta$)) are shown for CNN-DeepONet, FNN-DeepONet, UNet, and LNO trained on (b) dilatation heat maps only and (c) dilatation and distensibility heat maps.

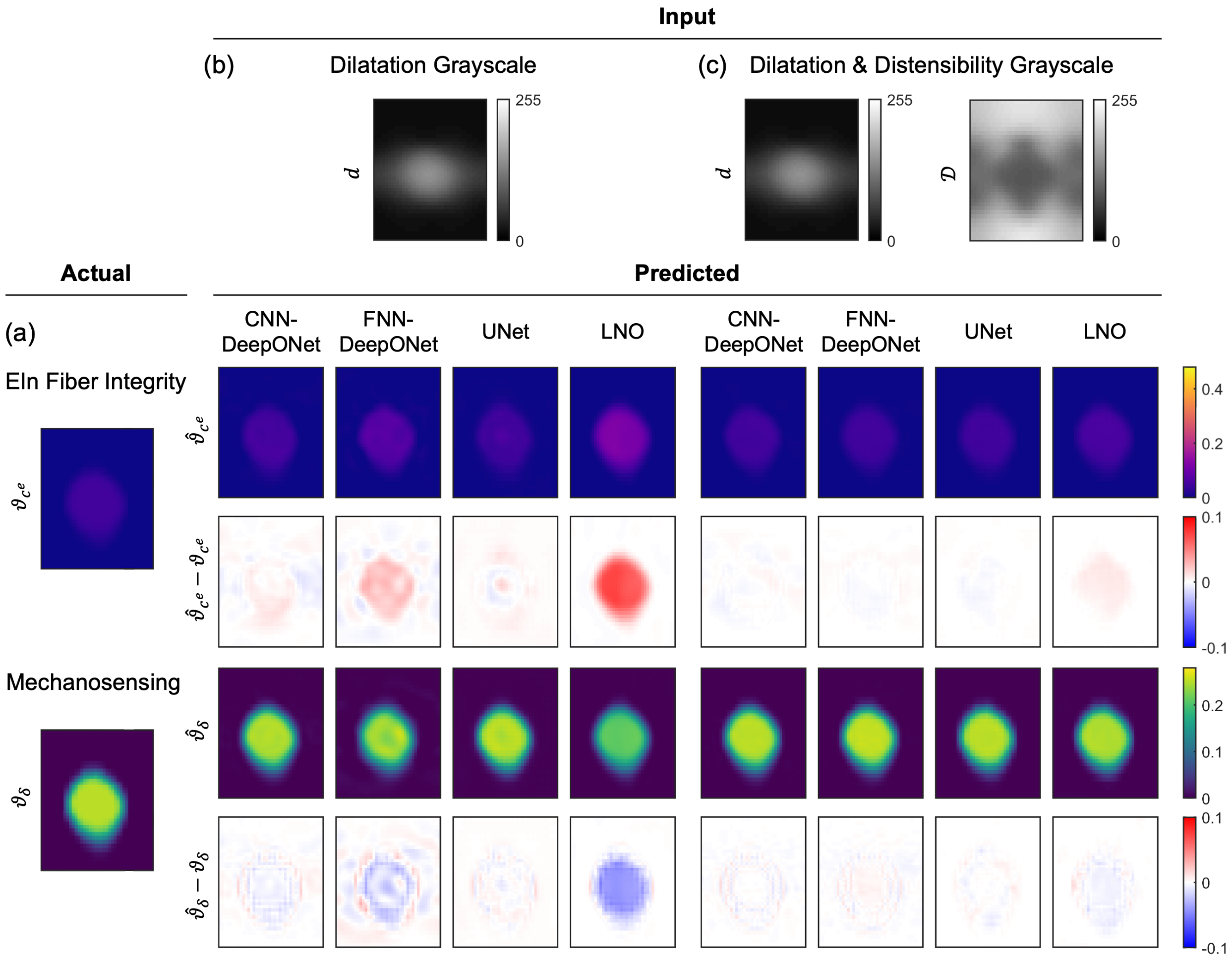

Fig D: Predictions from all four architectures for a mechanosensing-dominated combined insult. Similar to Figure C except for grayscale input maps. (a) The ground truth combined-insult field consisted of contributions of both compromised elastic fiber integrity (ϑ_{c^e}) and dysfunctional mechanosensing (ϑ_δ) superimposed in the FE simulation to generate the TAA with dilatation and distensibility profiles shown in (b–c). Predictions $\hat{\vartheta}_i$ and absolute errors $\hat{\vartheta}_i - \vartheta_i$ ($i = c^e, \delta$) are shown for CNN-DeepONet, FNN-DeepONet, UNet, and LNO trained on (b) dilatation grayscale maps only and (c) dilatation and distensibility grayscale maps.

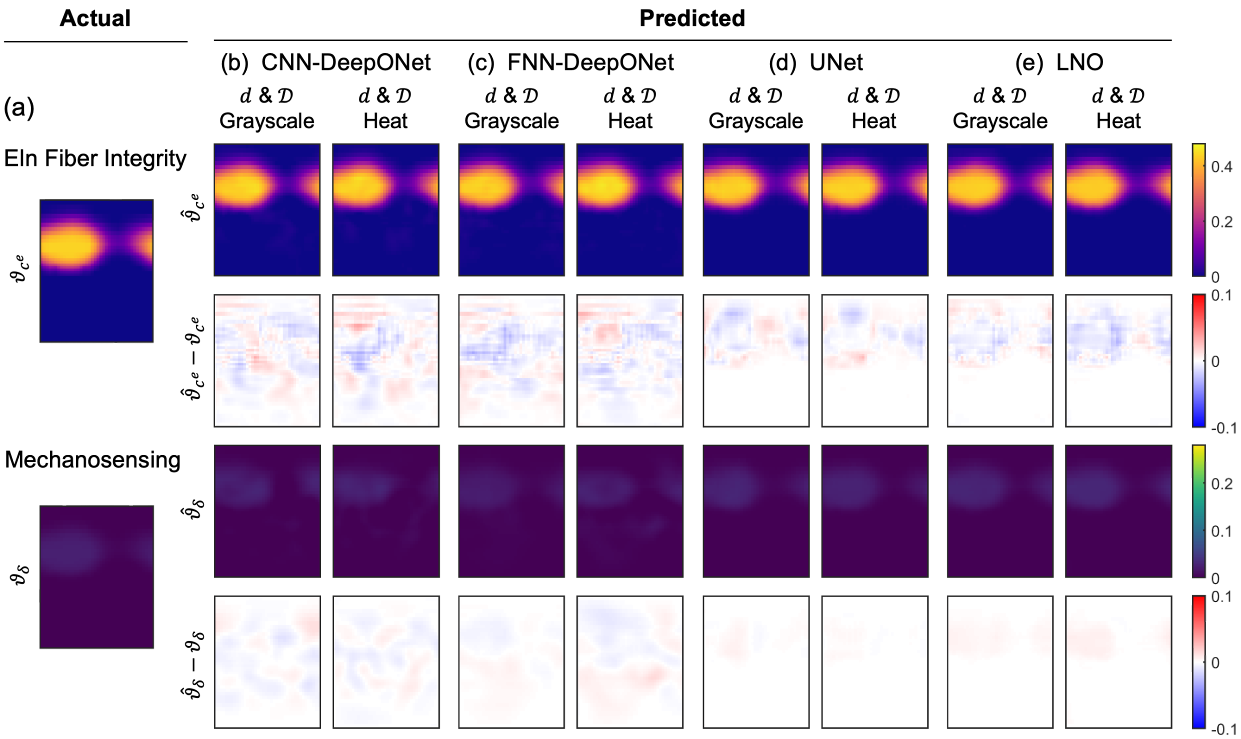

Fig E: Effects of grayscale and heat map data inputs. All networks were trained on dilatation (d) and distensibility ($\mathcal{D}$) maps. (a) Ground truths for elastic fiber integrity and mechanosensing contributions. Predictions ($\hat{\vartheta}_i$) and computation of absolute errors ($\hat{\vartheta}_i - \vartheta_i$ ($i = c^e, \delta$)) are shown for (b) CNN-DeepONet, (c) FNN-DeepONet, (d) UNet, and (e) LNO.

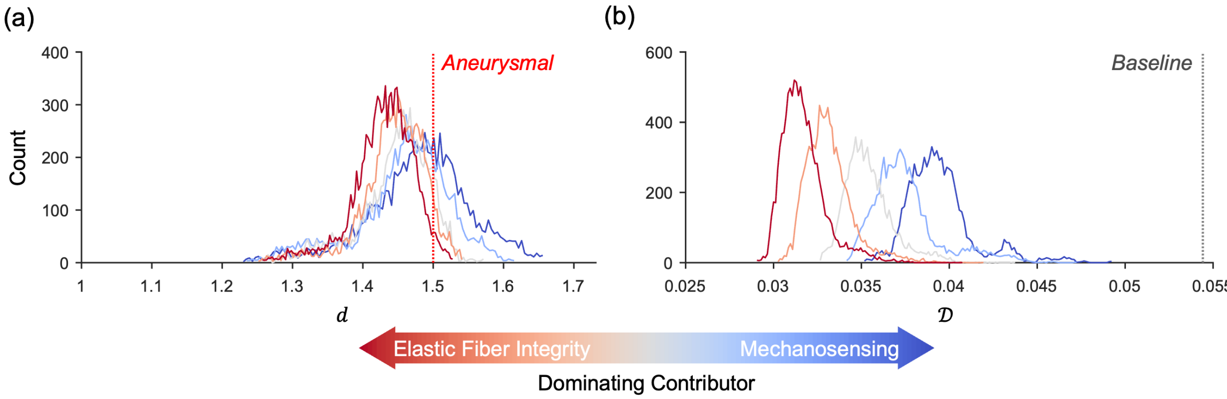

Fig F: Effects of insult contributor on dilatation and distensibility. Histograms of all pairs of (a) maximum dilatation and (b) minimum distensibility over all points within the insult region in 500 synthetic TAAs. Points are colored based on dominating combined insult contributor (spectrum shown at bottom). Aneurysmal dilatations are indicated as $d > 1.5$. Baseline distensibility (no insult applied) is indicated by the dotted gray line.

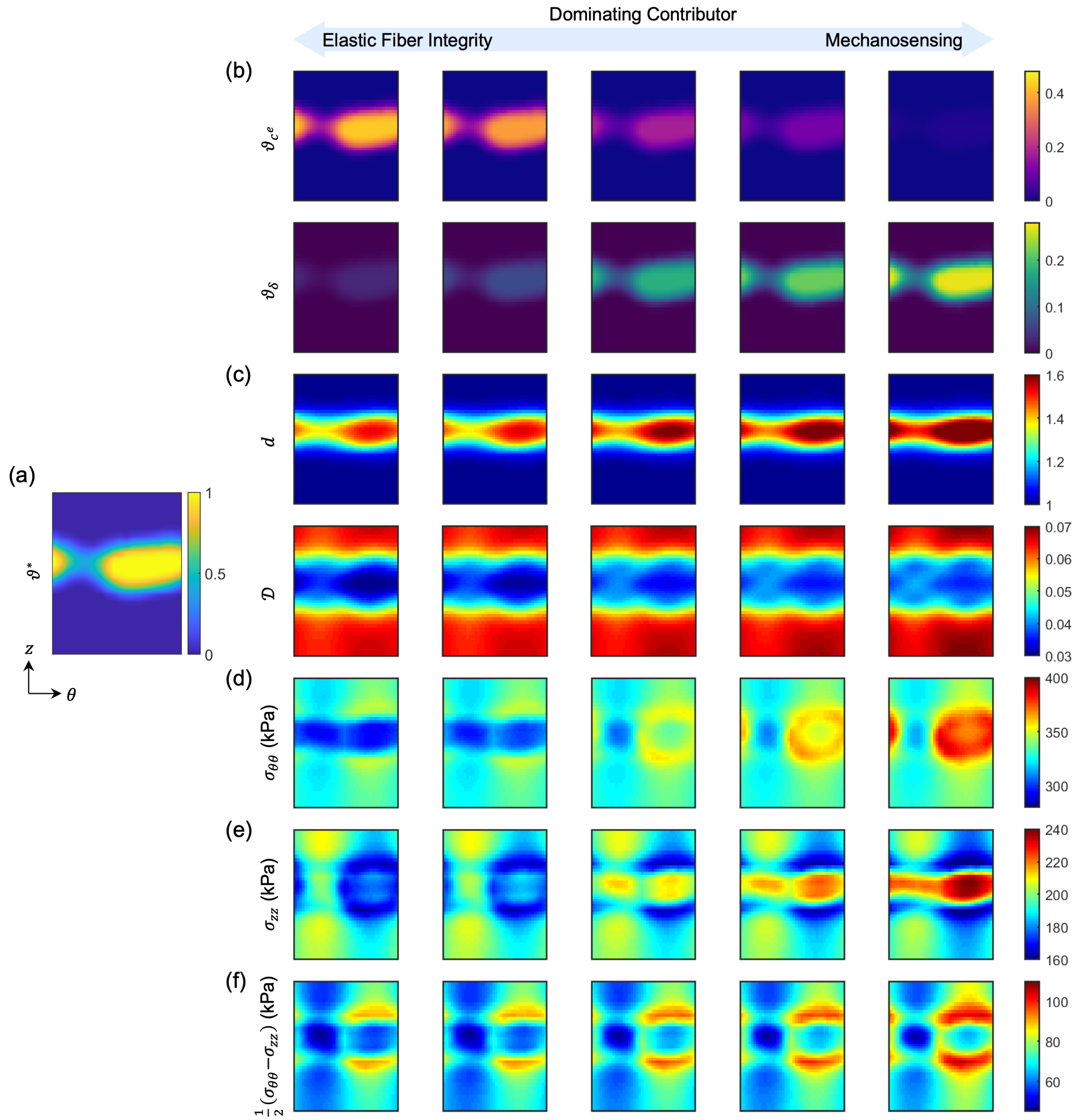

Fig G: Effects on geometry and mechanical properties. Similar to Figure A but with a different insult profile. (a) Normalized insult field (ϑ^*). (b) Degree of compromised integrity of elastic fibers ($\vartheta_{c^e} \in [0, 0.48]$, decreasing left-to-right) and dysfunctional mechanosensing ($\vartheta_\delta \in [0, 0.28]$, increasing left-to-right). (c) Heat maps for dilatation and distensibility for each combined insult. (d) Circumferential and (e) axial components of the Cauchy stress. (f) Estimated intramural shear stress computed as $(\sigma_{\theta\theta} - \sigma_{zz})/2$. Stress estimations underscore that the mechanobiological mechanisms driving dilatation may have a profound impact on the mechanical stability of the aneurysm and yet may appear nearly identical based on geometry alone.

References

[1] Murtada SI, Kawamura Y, Li G, Schwartz MA, Tellides G, Humphrey JD. 2021 Developmental origins of mechanical homeostasis in the aorta. Dev. Dyn. 250, 629–639. doi:10.1002/dvdy.283.

[2] Humphrey JD. 2021 Constrained mixture models of soft tissue growth and remodeling – twenty years after. J. Elast. 145, 49–75. doi:10.1007/s10659-020-09809-1.

[3] Cavinato C, Chen M, Weiss D, Ruiz-Rodríguez MJ, Schwartz MA, Humphrey JD. 2021 Progressive microstructural deterioration dictates evolving biomechanical dysfunction in the Marfan aorta. Front. Cardiovasc. Med. 8, 800730. doi:10.3389/fcvm.2021.800730.

[4] Li DS, Cavinato C, Latorre M, Humphrey JD. 2023 Computational modelling distinguishes diverse contributors to aneurysmal progression in the Marfan aorta. Proc. Royal Soc. A: Math. Phys. Eng. Sci. 479, 20230116. doi:10.1098/rspa.2023.0116.

[5] Goswami S, Li DS, Rego BV, Latorre M, Humphrey JD, Karniadakis GE. 2022 Neural operator learning of heterogeneous mechanobiological insults contributing to aortic aneurysms. J. The Royal Soc. Interface 19, 20220410. doi:10.1098/rsif.2022.0410.

[6] Latorre M, Humphrey JD. 2018 Modeling mechano-driven and immuno-mediated aortic maladaptation in hypertension. Biomech. Model. Mechanobiol. 17, 1497–1511. doi:10.1007/s10237-018-1041-8.
